# Supplementary material for: First Report of a Psyllid Vector of ‘Candidatus Phytoplasma pruni’ (Strain 16SrIII-J)
Source: Plants (Basel). 2025 Apr 23;14(9):1279. doi: 10.3390/plants14091279 (PMC12073468; doi:10.3390/plants14091279)
Supplement: Supplementary file 1 [file plants-14-01279-s001.zip › plants-3496753-supplementary.pdf]

SUPPLEMENTARY TABLES

Table S1. Information on the sequences in the phylogenetic tree elaborated with the *COI* gene.

Number	Species	GenBank Accession Number	Reference
1	*Russelliana* sp.	MG988824.1	[62]
2	*Russelliana solanicola*	NC_038140.1	[62]
3	*Dyspersa cerastii*	MT021816.1	[63] (as *Trioza cerastii*)
4	*Cacopsylla maculatili*	MK039629.1	[64]
5	*Cacopsylla picta*	OR351165.1	[65]
6	*Dyspersa pallida*	MG924938.1	Direct submission (as *Trioza anthrisci*)
7	*Pariaconus dorsostriatus*	PQ124090.1	Direct submission
8	*Drepanoza fruticulosi*	OR027204.1	[66]
9	*Cacopsylla burckhardti*	MK039638.1	[64]
10	*Dyspersa apicalis*	MG924936.1	Direct submission (as *Trioza apicalis*)
11	*Trioza urticae*	NC_038113.1	[62]
12	*Cacopsylla falcicauda*	OR027237.1	[66]
13	*Acizzia jamatonica*	MK039641.1	[64]
14	*Arytaina devia*	OR027236.1	[66]
15	*Lauritrioza laurisilvae*	OR027221.1	[66]
16	*Psyllopsis discrepans*	MT980798.1	[67]
17	*Cacopsylla citrisuga*	NC_053749.1	Direct submission
18	*Cacopsylla fraudatrix*	JX987969.1	[68]
19	*Cacopsylla myrtilli*	KR030733.1	[69]

Table S2. Information about the sequences used in the phylogenetic tree elaborated with mitochondrial genome.

Number	Species	GenBank Accession Number	Reference
1	*Russelliana solanicola*	NC_038140	[62]
2	*Trioza urticae*	NC_038113	[62]
3	*Dyspersa pallida*	NC_038141	[63] (as *Trioza anthrisci*)
4	*Cacopsylla picta*	OR346839	[62]
5	*Cyamophila willieti*	MN364946	[70]
6	*Cacopsylla burckhardti*	OK574466	[71]
7	*Cacopsylla coccinea*	NC_027087	[72]
9	*Acizzia uncatoides*	NC_038146	[62]
11	*Heteropsylla sp.*	NC_038149	[62]
12	*Aacanthocnema dobsoni*	NC_038132	[62]
13	*Arytainilla spartiophila*	NC_038133	[62]
14	*Freysuila caesalpiniae*	NC_038135	[62]
15	*Cacopsylla pyri*	NC_038148	[62]
16	*Cacopsylla jukyungi*	NC_069847	[73]
17	*Bactericera cockerelli*	NC_030055	[74]
18	*Psylla alni*	NC_038139	[62]
19	*Diaphorina citri*	MW021147	[75]

21	*Egeirotrioza xingi*	NC_087869	Direct submission
22	*Pariaconus minutus*	PQ124093	Direct submission
23	*Egeirotrioza gracilis*	PP471964	Direct submission
25	*Allocarsidara bakeri*	NC_038107	[62]
27	*Livia junci*	NC_038137	[62]
28	*Euphyllura phillyreae*	NC_038134	[62]
29	*Pachypsylla venusta*	AY278317	[76]
30	*Paracarsidara gigantea*	NC_038112	[62]
31	*Bactericera gobica*	NC_024577	[77] (as *Poratrioza sinica*)
32	*Leptynoptera sulfurea*	NC_038136	[62]
33	*Populicerus populi*	NC_039427	[78]
